# Functional impact and quality of life after total hip arthroplasty for hip osteoarthritis. A retrospective study of 200 cases.

**DOI:** 10.12688/f1000research.168896.1

**Published:** 2025-08-15

**Authors:** Aymen Fekih, Jacem Saadana, Firas Boughattas, Ikram Haddada

**Affiliations:** 1 Department of Orthopedic Surgery, Fattouma Bourguiba University Hospital of Monastir, Monastir, Tunisia; 2 Department of Physical Medicine and Rehabilitation, Taher Sfar University Hospital, Mahdia, Tunisia

**Keywords:** Osteoarthritis, Total hip arthroplasty, Functional impact, Quality of life

## Abstract

**Background:**

Total hip arthroplasty (THA) is now a well-established and reliable procedure that effectively reduces pain, restores mobility and enhances functionality. This study aim to evaluate the quality of life and functional outcomes of patients who underwent THA for hip osteoarthritis, and to identify the factors influencing these outcomes.

**Methods:**

A retrospective study was conducted involving 200 patients who underwent THA for hip osteoarthritis. Functional outcomes were assessed using the Postel-Merle d’Aubigné (PMA) score. Quality of life and patient satisfaction were evaluated using the Short Form Health Survey-12 (SF-12), the Hip Disability and Osteoarthritis Outcome Score (HOOS), the Pittsburgh Sleep Quality Index (PSQI), and the Hospital Anxiety and Depression Scale (HADS).

**Results:**

The PMA score increased from 7 to 14 points (p = 0.008), the SF-12 PCS rose from 29.76 to 48.71 (p < 0.001), and the SF-12 MCS increased from 49.24 to 54.49 (p < 0.001). The HOOS improved from 35/100 to 76/100 (p < 0.001). The PSQI decreased from 7/21 to 3/21 (p < 0.001). The HADS depression and anxiety fell from 6.8/21 to 3.8/21 (p < 0.001) and from 8.4/21 to 4.8/21 (p < 0.001), respectively. Physical and mental health were positively associated with walking autonomy and higher BMI. Hip function outcomes were better in patients with ceramic on-ceramic articulations, younger age, and higher postoperative PMA scores. Good sleep quality and decreased depression score were associated with increased walking distance and BMI. There were no significant associations between anxiety and any of the variables investigated.

**Conclusions:**

These findings highlight the effectiveness of THA not only in restoring mobility but also in enhancing overall patient well-being. Integrating both functional and quality-of-life assessments into patient care offers a comprehensive approach to postoperative management and may optimize long-term outcomes for patients with hip osteoarthritis.

## Introduction

Hip osteoarthritis is the primary condition leading to total hip arthroplasty (THA), a well-known procedure that has changed its treatment forever. ^1, 2^ THA aims to relieve pain, improve movement, restore function, and ultimately optimize the socio-professional life and quality of life of the affected patients. ^3^

Quality of life evaluation becomes the major criterion in the determination of THA’s efficacy. Such assessments are crucial for understanding patient needs, setting expectations, and evaluating treatment outcomes. Quality-of-life evaluations should encompass physical, psychological, and social domains. ^4^

This study aims to 1) evaluate the quality of life of patients undergoing THA for hip osteoarthritis using established, generic instruments: the Postel-Merle d’Aubigné score (PMA), the Short Form Health Survey (SF-12), the Hip Disability and Osteoarthritis Outcome Score (HOOS), the Pittsburgh Sleep Quality Index (PSQI), and the Hospital Anxiety and Depression Scale (HADS); and 2) analyze the factors that may influence postoperative quality of life in these patients.

## Materials and methods

### Study design

Approval was obtained from the Institutional Review Board (IRB) of Fattouma Bourguiba Hospital (CEM-2018-12-15) for the conduct of this retrospective study performed on patients who underwent primary THA for hip osteoarthritis from January 2019 to December 2020 at Fattouma Bourguiba Hospital. The design and reporting were performed in accordance to the Declaration of Helsinki. The written informed consent was waived by the IRB due to the retrospective nature of the study. Patient confidentiality was maintained through anonymized data collection and analysis.

The inclusion criteria for the patient’s analysis were: (1) patients undergoing THA for hip osteoarthritis, whether degenerative or secondary to a systemic disease; (2) patients capable of providing written or verbal responses to questionnaires; and (3) those with minimum follow-up duration of two years postoperatively.

The following patients were excluded: (1) post-traumatic THA cases; (2) patients with active infections or acute exacerbations of chronic medical conditions; (3) patients unable to complete self-reported questionnaires due to cognitive impairment; and (4) patients with significant neuromuscular or vascular comorbidities affecting lower limb function.

### Data collection protocol

We systematically collected data from electronic medical records using a standardized case report form that included (1) demographic and epidemiological data (age, sex, body mass index (BMI), occupational status, comorbidities, preoperative functional status), (2) clinical parameters (affected side and symptom duration, gait perimeter assessment, surgical approach, implant characteristics) and (3) postoperative evolution (immediate and late complications, rehabilitation protocol details). The scoring was based on a direct interview with the patient (190 cases) during follow-up and in rare cases (10 patients) it was based on a telephone interview with clarification of contentious issues.

### Functional evaluation

We assessed hip function using the validated Postel-Merle d’Aubigné (PMA) score preoperatively and at the final follow-up. This 18-point scale evaluates pain (0-6 points), mobility (0-6 points), and walking ability (0-6 points). ^5^ Scores were categorized as Very good (≥12 points), Good (10-11 points), Average (9 points), Low (8 points) and Poor (≤7 points).

### Quality of life assessment

We employed four validated instruments:

1) SF-12 Health Survey: assessed physical (PCS) and mental (MCS) health components and scored from 0 to 100. Higher scores indicate a better quality of life. ^6^
2) Hip Disability and Osteoarthritis Outcome Score: evaluated five domains: Pain (10 items), Symptoms (5 items), Activities of Daily Living (17 items), Sports and Recreation (4 items), and Hip-related quality of life (4 items). ^7^ Transformed to a 0-100 scale, higher scores indicate better function and less pain. ^8^
3) Pittsburgh Sleep Quality Index: used for assessing seven sleep components with a global score ranging from zero to 21. ^9^ A score greater than 5 indicates poor sleep quality. ^10^
4) Hospital Anxiety and Depression Scale: used for identifying and quantifying symptoms of anxiety and depression and divided into two subscales Anxiety subscale (HADS-A, 7 items) and Depression subscale (HADS-D, 7 items). Each subscale is scored from 0 to 21. ^11, 12^ The following cutoff scores are commonly used to interpret the HADS subscale scores: 0-7: Normal; 8-10: Mild symptoms and 11-21: Moderate to severe symptoms. ^13^

### Statistical analysis

We performed all analyses using SPSS version 26 (IBM Corp.). Descriptive statistics included means for normally distributed continuous variables, medians for non-normal distributions, and frequencies for categorical variables. For inferential analysis, we used Student’s t-test for normally distributed continuous variables, Pearson’s chi-square or Fisher’s exact test for categorical variables, and Pearson correlation for continuous variable associations. All tests were two-tailed with a p = 0.05 significance threshold.

## Results

### Study population and baseline characteristics

This study included 200 eligible patients. The cohort had a male predominance (59% male, 41% female), with a male-to-female ratio of 1.43. The mean age was 49 ± 15.59 years (range: 35–78), with most patients (62.5%) aged between 40 and 65 years. The right hip was operated on 120 patients and the left hip on 80. THA was bilateral in 30 patients ( Table 1).

**Table 1.** Characteristics of the population’s study.

Population characteristics	Total (n=200)
**Sex**	
Male	108 (59)
Female	82 (41)
**Age (years)**	
<40	25 (12.5)
[40-65]	125 (62.5)
≥65	50 (50)
**Operated side**	
Right	120 (60)
Left	80 (40)
**BMI ((kg/m** ^**2**^ **)**	
Normal (18.5 to 24.9)	82 (41)
Overweight (25 to 29.9)	78 (39)
Obese (≥30)	40 (20)
**Occupational status**	
Employees	102 (51)
Housewives	48 (24)
Retired	50 (25)
**Causes of hip osteoarthritis**	
Primary	105 (52.5)
Rheumatoid arthritis	65 (32.5)
Ankylosing spondylitis	30 (15)
**Friction torque**	
Ceramic on ceramic	120 (60)
Metal on highly cross-linked polyethylene	80 (40)
**Femoral stem**	
Cemented	74 (37)
Uncemented	126 (63)
**Acetabular component**	
Cemented	76 (38)
Uncemented	124 (62)
**Postoperative walk autonomy**	
Independent	165 (82.5)
Single cane	27 (13.5)
Two canes	8 (4)

Values are presented as number (%). BMI, body mass index.

BMI distribution revealed that 20% of patients were obese, 39% overweight, and 41% had a normal BMI. Occupational status varied: 51% were employees, 24% were housewives, and 25% were retired. Metabolic syndrome was present in 30% of the cohort.

The average duration of symptoms was four years, with extremes ranging from two to six years. Primary hip joint involvement was observed in 52.5% of cases, while 47.5% were secondary to systemic disease mainly rheumatoid arthritis and ankylosing spondylitis. All patients presented with a reduced gait perimeter, with a median walking distance of 200 ± 158 meters preoperatively.

In all cases, the surgical approach used was the posterolateral Moore technique. Ceramic on-ceramic articulation was used in 60% of cases. Metal on highly cross-linked polyethylene was used in 40% of cases. Regarding prosthesis fixation, femoral stems were cemented in 37% of cases, while 63% were uncemented. These ratios are similar for acetabular components (38% cemented and 62% uncemented).

Five cases of prosthesis dislocation occurred within two months of surgery, with no recurrence. There were also ten cases of early sepsis, which were managed through repeat surgery involving debridement and lavage, as well as appropriate antibiotic therapy. The outcome was good. No late complications were reported at the last follow-up.

Postoperatively, all patients stood up on the day of the operation, and 75% began ambulating immediately the day after. Rehabilitation protocol applied to all patients included muscle strengthening, analgesic physiotherapy, and patient education to prevent dislocating movements of the prosthesis.

After a mean follow-up of three years (range: two to four years), 82.5% walked independently, 13.7% used a single cane, and only 3.8% required two canes, demonstrating significant functional improvement (p = 0.017).

### Functional and quality of life outcomes

THA led to significant functional improvements ( Table 2), as demonstrated by the PMA scale (p = 0.008). The mean PMA score more than doubled, increasing from 7/18 (±2) preoperatively to 14/18 (±4) postoperatively. Clinically, this translated to a dramatic decline in patients with “poor” function (53.8% preoperatively vs. postoperatively) and a substantial rise in those achieving “very good” scores (87.2%).

**Table 2.** Functional and quality of life outcomes.

	Preoperatively	Postoperatively
**PMA score**		
Poor	53.8%	0
Low	28.2%	0
Average	14.1%	0
Good	3.8%	12.8%
Very good	0	87.2%
**SF-12 score**		
PCS	29.76	48.71
MCS	49.24	54.49
**HOOS**		
Symptoms	50	87.5
Pain	35	80
Activity	37.5	80.9
Sports and recreation	18.8	43.8
Quality of life	31.3	68.8
**PSQI**		
Bad sleepers	75.6%	12.8%
Good sleepers	24.4%	87.2%
**HADS**		
Definite depression	12.8%	9%
No depression	87.2%	91%
Definite anxiety	28.2%	5.1%
No anxiety	71.8%	94.9%

PMA, Postel-Merle d'Aubigné; SF-12, short form health survey-12; HOOS, hip disability and osteoarthritis outcome; PSQI, Pittsburgh sleep quality index; HADS, hospital anxiety and depression scale.

Patients also experienced marked enhancements in quality of life, measured by the SF-12 survey. The PCS improved from 29.76 to 48.71 (p < 0.001), while the MCS rose from 49.24 to 54.49 (p < 0.001), indicating better physical and psychological well-being.

Hip-specific function, assessed via the HOOS, showed significant gains (p < 0.001), with the global score improving from 35 to 76. Preoperatively, the greatest limitations were in Sports/Leisure activities, while the Symptoms domain was the least severe among the HOOS subscales. Postoperatively, Activity domains improved the most, though Sports/Leisure remained challenging.

Sleep quality, evaluated using the PSQI, also improved significantly, with scores dropping from 7/21 (±2.75) to 3/21 (±2.07) (p < 0.001). The proportion of “good sleepers” (PSQI ≤5) increased from 24.4% to 87.2%.

Psychological assessments using the HADS revealed reduced depression from 6.8/21 ±2.86 to 3.8/21 ±2.21 (p < 0.001) and anxiety from 8.4/21 ±3.06 to 4.8/21 ±2.66) (p < 0.001). Preoperatively, 12.8% had “definite depression” and 28.2% had “definite anxiety”; postoperatively, these rates fell to 9% and 5.1%, respectively.

### Factors influencing postoperative quality of life after total hip arthroplasty

This study identified several significant factors associated with improved quality of life following THA ( Table 3). Physical health outcomes (SF-12 PCS) showed positive correlations with a history of rheumatic diseases (p = 0.017), post-operative walking autonomy (p = 0.011), shorter hospital stay (p = 0.048), and earlier mobilization (p = 0.046). Mental health (SF-12 MCS) was significantly linked to walking autonomy (p = 0.011), greater walking distance (p = 0.003), and BMI (p = 0.043).

**Table 3.** Factors influencing postoperative quality of life after total hip arthroplasty.

	Postoperative PCS	p-value	Postoperative MCS	p-value	Postoperative HOOS	p-value
** Sex**		0.683		0.412		0.944
Male (n=108)	48.45±6.90		54.04±6.11		75.59±9.35	
Female (n=82)	49.07±6.38		55.16±5.23		75.44±8.35	
**Age (years)**		0.053		0.053		**0.002**
≤40	51.65±6.52		56.45±5.03		79.42±8.97	
>40	46.79±4.67		54.63±4.73		68.28±7.56	
**Operated side**		0.485		0.476		0.773
Unilateral (n=170)	49.20±5.67		54.05±0.88		75.80±9.16	
Bilateral (n=30)	48.13±7.69		55.01±1.02		75.21±8.70	
**BMI**	49.24±6.44	0.906	54.72±5.87	**0.043**	76.18±8.68	0.592
**Rheumatic diseases**		**0.017**		0.403		0.091
No (n=105)	46.56±6.58		53.66±6.03		72.58±8.28	
Yes (n=95)	50.43±5.11		54.98±5.77		76.78±10.17	
**Friction torque**		0.160		0.631		**0.03**
Metal on highly cross-linked polyethylene (n=80)	48.21±6.79		54.31±6.06		73.97±9.12	
Ceramic on-ceramic (n=120)	50.49±6.20		54.99±5.48		78.67±8.40	
**Mobilization**	48.82±6.81	**0.046**	54.62±5.82	0.874	75.79±8.79	**0.027**
**Hospital stay**	49.20±6.29	**0.048**	54.43±5.91	0.933	75.83±8.94	0.229
**Postoperative walk autonomy**		**0.011**		**0.011**		0.352
Independent (n=165)	49.59±6.44		54.68±5.55		77.79±7.88	
Single or two canes (n=35)	46.83±6.12		53.59±6.75		66.45±5.72	
**Walking distance**	48.66±6.82	0.083	54.50±5.80	**0.003**	75.73±8.54	0.541
**Postoperative PMA**	48.52±1.90	0.501	54.00±2.38	0.450	75.53±1.90	**0.01**

PCS: physical component summary, MCS: mental component summary, HOOS: hip disability and osteoarthritis outcome, BMI: body mass index, PMA: Postel-Merle d'Aubigné.

Hip function, assessed by the HOOS, improved with ceramic on ceramic articulation (p = 0.03), younger age (p = 0.002), earlier post-operative mobilization (p = 0.027), and higher post-operative PMA scores (p = 0.01). Sleep quality was positively associated with walking distance (p = 0.018), while depression scores decreased with increased walking distance (p = 0.037) and higher BMI (p = 0.043). No significant associations were found between anxiety and any of the studied variables (p > 0.05).

## Discussion

This study, involving 200 patients with hip osteoarthritis, investigated the impact of THA on functional and quality of life outcomes. We found significant improvements across multiple domains post-THA.

Functional outcomes, assessed by the PMA score, more than doubled from a mean of 7 pre-operatively to 14 post-operatively. This aligns with existing literature demonstrating a clear improvement in PMA scores, rising from 6.9 preoperatively to 15 postoperatively. ^14, 15^

Quality of life was significantly enhanced, as evidenced by validated tools such as SF-12, HOOS, PSQI, and HAD scores. ^16^

The SF-12 PCS increased from 29.76 to 48.71, while the MCS rose from 49.24 to 54.49. These findings are consistent with previous research demonstrating a statistically significant improvement in mean PCS (31.2 ± 9.7, p = 0.001) and MCS (51.6 ± 17.6, p = 0.024) after THA. ^17^ Several studies have concluded that patients’ quality of life automatically improves with improved hip function, based on functional scores. ^18^

The HOOS, a hip-specific questionnaire, ^19^ showed a marked improvement from 35 pre-operatively to 76 post-operatively across all domains, including the most affected pre-operative domains: HOOS-Sports and Recreation and HOOS-Quality of Life. However, one study noted no improvement in Sports and Recreation. ^20^

Sleep quality significantly improved, with the overall PSQI decreasing from 7/21 to 3/21. The percentage of “good sleepers” increased from 24.4% to 87.2%. Postoperative sleep quality depends on several factors, some of which are uncontrollable, such as advanced age and preoperative comorbidities, while others are controllable, such as the invasiveness of the surgical procedure, the type of anesthesia, and postoperative pain that prevents the patient from finding a comfortable sleeping position. ^21^ Pre-operative pain severity and duration are known risk factors for postoperative pain and sleep issues. ^22^

Psychological distress also decreased significantly. The HAD score showed a reduction in total depression scores from 6.8/21 to 3.8/21, and anxiety scores from 8.4/21 to 4.8/21. This led to a decrease in “definite depression” from 12.8% to 9% and “definite anxiety” from 28.2% to 5.1%. Pre-operative screening and treatment for depression and anxiety can optimize outcomes and reduce costs. ^23^ While psychological disorders aren’t contraindications, their treatment can improve outcomes, as painful complaints post-THA can have a psychiatric origin. ^24, 25^

Several factors positively influenced post-operative quality of life such as history of rheumatic diseases, walking autonomy, shorter hospital stays, earlier mobilization, ceramic-on-ceramic articulation, younger age, and higher BMI.

These findings underscore the critical role of mobility, early rehabilitation, and appropriate implant selection in achieving optimal recovery and overall well-being after THA.

This study’s limitations include its retrospective data analysis, the absence of a control group, and the lack of analysis regarding potential confounding factors. Specifically, the impact of pain management, socioeconomic status, and surgeon experience on outcomes was not assessed, which could influence the generalizability and interpretation of the findings.

## Conclusion

Total Hip Arthroplasty significantly improves the quality of life for patients with hip osteoarthritis by restoring mobility and enhancing overall well-being. To further optimize care, future efforts should focus on personalized rehabilitation, enhanced preoperative assessments, and integrated mental health support. This will address persistent limitations and psychological symptoms, solidifying THA’s value as a holistic treatment.

## Ethical considerations

Approval was obtained from the Institutional Review Board (IRB) of Fattouma Bourguiba Hospital (CEM-2018-12-15) for the conduct of the study.

The design and reporting were performed in accordance to the Declaration of Helsinki. The written informed consent was waived by the IRB due to the retrospective nature of the study. Patient confidentiality was maintained through anonymized data collection and analysis.

## Data Availability

The datasets generated and/or analysed during the current study are not publicly available due to preserve data confidentiality and patient anonymity but are available from the corresponding author Dr Fekih Aymen (e-mail: Fekih.aymen@gmail.com) on reasonable request. The protection of personal data is governed by a law promulgated by the National Authority for Protection of Personal Data in Tunisia which stipulates that data may only be shared after informed consent and request. There is no intermediary data that can be de-identified without compromising anonymity. The same thing was explained by the Institutional Review Board of our hospital.

## References

[1] Fautrel B Hilliquin P Rozenberg S : Impact of osteoarthritis: results of a nationwide survey of 10,000 patients consulting for OA. *Joint Bone Spine.* 2005;72:235–240. 10.1016/j.jbspin.2004.08.009 15850995

[2] Belbachir A : Prise en charge de la douleur après prothèse totale de hanche. *Douleurs Eval. - Diagn. - Trait.* 2012;13:63–73. 10.1016/j.douler.2012.02.009

[3] Kassimi EH Abdelfettah Y Khadir A : Résultats fonctionnels et qualité de vie après prothèse totale de hanche: à propos de 93 cas. *J. Réadapt. Médicale Prat. Form. En Médecine Phys. Réadapt.* 2014;34:60–65. 10.1016/j.jrm.2014.03.003

[4] Currey HL : Osteoarthrosis of the hip joint and sexual activity. *Ann. Rheum. Dis.* 1970;29:488–493. 10.1136/ard.29.5.488 5476676 PMC1010561

[5] d’Aubigné RM Postel M : The Classic: Functional Results of Hip Arthroplasty with Acrylic Prosthesis. *Clin. Orthop. Relat. Res.* 2009;467:7–27. 10.1007/s11999-008-0572-1 18941852 PMC2600978

[6] Younsi M Chakroun M : Measuring health-related quality of life: psychometric evaluation of the Tunisian version of the SF-12 health survey. *Qual. Life Res.* 2014;23:2047–2054. 10.1007/s11136-014-0641-8 24515673

[7] Lyman S Lee YY Franklin PD : Validation of the HOOS, JR: A Short-form Hip Replacement Survey. *Clin. Orthop. Relat. Res.* 2016;474:1472–1482. 10.1007/s11999-016-4718-2 26926772 PMC4868170

[8] Nilsdotter AK Lohmander LS Klässbo M : Hip disability and osteoarthritis outcome score (HOOS)-validity and responsiveness in total hip replacement. *BMC Musculoskelet. Disord.* 2003;4:10. 10.1186/1471-2474-4-10 12777182 PMC161815

[9] Suleiman KH Yates BC Berger AM : Translating the Pittsburgh Sleep Quality Index Into Arabic. *West. J. Nurs. Res.* 2010;32:250–268. 10.1177/0193945909348230 19915205

[10] Buysse DJ Reynolds CF Monk TH : The Pittsburgh sleep quality index: A new instrument for psychiatric practice and research. *Psychiatry Res.* 1989;28:193–213. 10.1016/0165-1781(89)90047-4 2748771

[11] Zigmond AS Snaith RP : The Hospital Anxiety and Depression Scale. *Acta Psychiatr. Scand.* 1983;67:361–370. 10.1111/j.1600-0447.1983.tb09716.x 6880820

[12] Herrmann C : International experiences with the Hospital Anxiety and Depression Scale-A review of validation data and clinical results. *J. Psychosom. Res.* 1997;42:17–41. 10.1016/S0022-3999(96)00216-4 9055211

[13] Terkawi A Tsang S AlKahtani G : Development and validation of Arabic version of the Hospital Anxiety and Depression Scale. *Saudi J. Anaesth.* 2017;11(Suppl. 1):S11–S18. 10.4103/sja.SJA_43_17 28616000 PMC5463562

[14] Meftah S Belhaj K Zahi S : Comparison of functional outcomes after total hip replacement surgery on degenerative and inflammatory disease in a Moroccan population. *J. Réadapt. Médicale Prat. Form. En Médecine Phys. Réadapt.* 2016;36:185–188.

[15] Chaminade B Laffosse JM Molinier F : Total hip arthroplasty in patients younger than 30-years-old: Results with activity level. *Rev. Chir. Orthop. Reparatrice Appar. Mot.* 2008;94(Suppl. 6):S173–S176. 18928810 10.1016/j.rco.2008.07.269

[16] Gandek B Ware JE Aaronson NK : Cross-validation of item selection and scoring for the SF-12 Health Survey in nine countries: results from the IQOLA Project. International Quality of Life Assessment. *J. Clin. Epidemiol.* 1998;5:1171–1178. 10.1016/s0895-4356(98)00109-7 9817135

[17] Bahardoust M Hajializade M Amiri R : Evaluation of health-related quality of life after total hip arthroplasty: a case-control study in the Iranian population. *BMC Musculoskelet. Disord.* 2019;20:46. 10.1186/s12891-019-2428-0 30704434 PMC6357390

[18] Ethgen O Bruyère O Richy F : Health-related quality of life in total hip and total knee arthroplasty. A qualitative and systematic review of the literature. *J. Bone Joint Surg. Am.* 2004;86:963–974. 15118039 10.2106/00004623-200405000-00012

[19] Shields RK Enloe LJ Leo KC : Health related quality of life in patients with total hip or knee replacement. *Arch. Phys. Med. Rehabil.* 1999;80:572–579. 10.1016/S0003-9993(99)90202-2 10326924

[20] Judd DL Dennis DA Thomas AC : Muscle strength and functional recovery during the first year after THA. *Clin. Orthop. Relat. Res.* 2014;472:654–664. 10.1007/s11999-013-3136-y 23817756 PMC3890211

[21] El Chalouhi M : *Sommeil et hypersensibilité préopératoires: comparaison entre les patients prévus pour une chirurgie élective prothétique de genou et de hanche. Faculté de médecine et médecine dentaire.* Université catholique de Louvain;2020. Prom.: Lavand’homme, Patricia. Reference Source

[22] Latremoliere A Woolf CJ : Central sensitization: a generator of pain hypersensitivity by central neural plasticity. *J. Pain.* 2009;10:895–926. 10.1016/j.jpain.2009.06.012 19712899 PMC2750819

[23] Mossey JM Knott K Craik R : The effects of persistent depressive symptoms on hip fracture recovery. *J. Gerontol.* 1990;45:M163–M168. 10.1093/geronj/45.5.M163 2394912

[24] Riediger W Doering S Krismer M : Depression and somatisation influence the outcome of total hip replacement. *Int. Orthop.* 2010;34:13–18. 10.1007/s00264-008-0688-7 19034446 PMC2899264

[25] Pacault-Legendre V Anract P Mathieu M : Pain after total hip arthroplasty: a psychiatric point of view. *Int. Orthop.* 2009;33:65–69. 10.1007/s00264-007-0470-2 17968546 PMC2899224

