# Supplementary material for: Functional impact and quality of life after total hip arthroplasty for hip osteoarthritis. A retrospective study of 200 cases
Source: F1000Res. 2025 Aug 15;14:792. [Version 1] doi: 10.12688/f1000research.168896.1 (PMC13348698; doi:10.12688/f1000research.168896.1)
Supplement: Peer review history

# Reviewer report: Reviewer response for version 1

**DOI:** 10.5256/f1000research.186116.r448064

**Published:** 2026-03-10

**Authors:** Mourad Jenzri

**Affiliations:** 1 MT Kassab Orthopaedic Institute, Tunis, Tunisia

Title: Functional impact and quality of life after total hip arthroplasty for hip osteoarthritis. A retrospective study of 200 cases.

 Reviewing assessment: **Approved**

**Executive Summary**

 The study investigates the functional and psychological outcomes of THA with a minimum two-year follow-up. Using a wide battery of validated tools (PMA, HOOS, SF-12, PSQI, and HADS), the authors demonstrate significant improvements in:

- **Physical Function:** Substantial gains in mobility and hip-specific scores.
- **Quality of Life:** Enhanced general health perception.
- **Well-being:** Notable reductions in sleep disturbances, anxiety, and depression.

**Key Strengths**

- **Holistic Assessment:** Moves beyond simple joint function to include mental health and sleep.
- **Sample Size:** 200 cases provide strong statistical power.
- **Real-World Data:** Reflects routine clinical practice, making findings highly generalizable.

**Recommendations for Improvement**

1. **Complication Analysis:** Compare outcomes of patients who suffered adverse events (infection/dislocation) against uncomplicated cases to see how "robust" the recovery is when things go wrong.
2. **Age Stratification:** Analyze results by age groups to account for differing physiological reserves and lifestyle expectations (e.g. return to work vs. simple pain-free walking).
3. **Etiology Focus:** Specifically explore the 47.5% of the cohort with secondary OA (systemic inflammatory disease), as this represents a novel contribution to existing literature.
4. **Long-term Outlook:** Explicitly state in the conclusion that while 2-3 year results are excellent, long-term follow-up is required to monitor potential degradation due to implant wear or aging.

Is the work clearly and accurately presented and does it cite the current literature?

Yes

If applicable, is the statistical analysis and its interpretation appropriate?

Yes

Are all the source data underlying the results available to ensure full reproducibility?

Yes

Is the study design appropriate and is the work technically sound?

Yes

Are the conclusions drawn adequately supported by the results?

Yes

Are sufficient details of methods and analysis provided to allow replication by others?

Yes

Reviewer Expertise:

The article shows the results of hip arthroplasty using functional scores with a record of complications (results that can be compared with other series).

I confirm that I have read this submission and believe that I have an appropriate level of expertise to confirm that it is of an acceptable scientific standard.

# Reviewer report: Reviewer response for version 1

**DOI:** 10.5256/f1000research.186116.r448067

**Published:** 2026-01-20

**Authors:** Aymen Ben Fredj

**Affiliations:** 1 IBN EL JAZZAR University Hospital, Kairouan, Tunisia

This retrospective study evaluates functional and quality of life outcomes in 200 patients who underwent total hip arthroplasty for hip osteoarthritis, with a minimum follow-up of two years. Using validated functional, physical, psychological, and sleep-related outcome measures (PMA, SF-12, HOOS, PSQI, and HADS), the authors demonstrate significant postoperative improvements across all assessed domains and identify factors associated with better outcomes.

 The manuscript is clearly written, well structured, and supported by relevant and up-to-date literature. The study design is appropriate for the research objectives, and the methodology is technically sound. The inclusion/exclusion criteria, outcome measures, and data collection procedures are adequately described, allowing reproducibility.

 The statistical analyses are appropriate and correctly interpreted. Results are presented clearly and support the conclusions drawn. Source data availability and ethical considerations are properly addressed.

 Strengths of this study include the relatively large sample size, use of multiple validated outcome instruments, and a comprehensive assessment of functional and quality-of-life outcomes.

 The limitations, notably the retrospective design and absence of a control group, are appropriately acknowledged and do not undermine the scientific validity of the study.

Is the work clearly and accurately presented and does it cite the current literature?

Yes

If applicable, is the statistical analysis and its interpretation appropriate?

Yes

Are all the source data underlying the results available to ensure full reproducibility?

Yes

Is the study design appropriate and is the work technically sound?

Yes

Are the conclusions drawn adequately supported by the results?

Yes

Are sufficient details of methods and analysis provided to allow replication by others?

Yes

Reviewer Expertise:

Hip and knee surgery, including total hip arthroplasty and surgical management of hip osteoarthritis

I confirm that I have read this submission and believe that I have an appropriate level of expertise to confirm that it is of an acceptable scientific standard.
